# Gene expression profile of intramuscular muscle in Nellore cattle with extreme values of fatty acid

**DOI:** 10.1186/s12864-016-3232-y

**Published:** 2016-11-25

**Authors:** Mariana P. Berton, Larissa F. S. Fonseca, Daniela F. J. Gimenez, Bruno L. Utembergue, Aline S. M. Cesar, Luiz L. Coutinho, Marcos Vinicius A. de Lemos, Carolyn Aboujaoude, Angélica S. C. Pereira, Rafael M. de O Silva, Nedenia B. Stafuzza, Fabieli L. B. Feitosa, Hermenegildo L. J. Chiaia, Bianca F. Olivieri, Elisa Peripolli, Rafael L. Tonussi, Daniel M. Gordo, Rafael Espigolan, Adrielle M. Ferrinho, Lenise F. Mueller, Lucia G. de Albuquerque, Henrique N. de Oliveira, Susan Duckett, Fernando Baldi

**Affiliations:** 1 Departamento de Zootecnia, Universidade Estadual Paulista, Faculdade de Ciências Agrárias e Veterinárias, Jaboticabal, 14884-900 SP Brazil; 2 Departamento de Nutrição e Produção Animal, Universidade de São Paulo, Faculdade de Medicina Veterinária e Zootecnia, Pirassununga, 13635-900 SP Brazil; 3 Departamento de Zootecnia, Universidade de São Paulo, Piracicaba, 13418-900 SP Brazil; 4 Departamento de Zootecnia, Universidade de São Paulo, Faculdade de Zootecnia e Engenharia de Alimentos, Pirassununga, 13635-900 SP Brazil; 5 Conselho Nacional de Desenvolvimento Científico e Tecnológico, Lago Sul, 71605-001 DF Brazil; 6 Animal and Veterinary Science Department of Clemson University, Clemson, 29634 SC USA

**Keywords:** *Bos indicus*, Human health, Lipid composition, RNA-seq, Transcriptome

## Abstract

**Background:**

Fatty acid type in beef can be detrimental to human health and has received considerable attention in recent years. The aim of this study was to identify differentially expressed genes in *longissimus thoracis* muscle of 48 Nellore young bulls with extreme phenotypes for fatty acid composition of intramuscular fat by RNA-seq technique.

**Results:**

Differential expression analyses between animals with extreme phenotype for fatty acid composition showed a total of 13 differentially expressed genes for myristic (C14:0), 35 for palmitic (C16:0), 187 for stearic (C18:0), 371 for oleic (C18:1, cis-9), 24 for conjugated linoleic (C18:2 cis-9, trans11, CLA), 89 for linoleic (C18:2 cis-9,12 n6), and 110 genes for α-linolenic (C18:3 n3) fatty acids. For the respective sums of the individual fatty acids, 51 differentially expressed genes for saturated fatty acids (SFA), 336 for monounsaturated (MUFA), 131 for polyunsaturated (PUFA), 92 for PUFA/SFA ratio, 55 for ω3, 627 for ω6, and 22 for ω6/ω3 ratio were identified. Functional annotation analyses identified several genes associated with fatty acid metabolism, such as those involved in intra and extra-cellular transport of fatty acid synthesis precursors in intramuscular fat of *longissimus thoracis* muscle. Some of them must be highlighted, such as: *ACSM3* and *ACSS1* genes, which work as a precursor in fatty acid synthesis; *DGAT2* gene that acts in the deposition of saturated fat in the adipose tissue; *GPP* and *LPL* genes that support the synthesis of insulin, stimulating both the glucose synthesis and the amino acids entry into the cells; and the *BDH1* gene, which is responsible for the synthesis and degradation of ketone bodies used in the synthesis of ATP.

**Conclusion:**

Several genes related to lipid metabolism and fatty acid composition were identified. These findings must contribute to the elucidation of the genetic basis to improve Nellore meat quality traits, with emphasis on human health. Additionally, it can also contribute to improve the knowledge of fatty acid biosynthesis and the selection of animals with better nutritional quality.

**Electronic supplementary material:**

The online version of this article (doi:10.1186/s12864-016-3232-y) contains supplementary material, which is available to authorized users.

## Background

Beef is characterized by its high nutritional value, being an important source of protein, essential amino acids, vitamins (A, B6, B12, D), and minerals such as iron, zinc and selenium [1, 2]. The fats present in beef are rich in long chain polyunsaturated fatty acid, which participate in several biological processes relevant to human health. In addition, beef fatty acids (FAs) composition plays an important role in the oxidative stability during the cooking process, affecting beef’s tenderness, flavor and juiciness [3]. Additionally, beef is a natural source of essential FAs, such as linoleic acid and conjugated linoleic acid (CLA) isomers, in particular the cis 9, trans 11 isomer and oleic acid [4–7]. Fatty acids type in beef, however, can have detrimental effect on human health if consumed in large quantities due to lipid composition, which is predominantly composed by saturated fatty acids (SFA) and has been associated with obesity, cardiovascular diseases and high cholesterol rate [8].

The major factors that influence beef FAs composition are animal age, diet, and breed type. Several studies have demonstrated that intramuscular fat from *Bos indicus* breeds is less saturated than those from *Bos taurus* [9–13]. In this regard, [13] pointed out that Nellore beef is nutritionally healthier than Angus beef, since it has lower percentages of cholesterol and higher amounts of ω3 FA and CLA precursor (C18:1 *trans*). Bressan et al. [14] comparing *Bos taurus* and *Bos indicus* animals showed that the production system has an important role on beef’s FAs composition. These authors reported that *Bos taurus* animals had lower percentage of SFA and higher percentage for monounsaturated fatty acids (MUFA) than *Bos indicus* animals finished in feedlot. According to them, *Bos taurus* cattle finished under feedlot conditions have higher ability to desaturate SFA than *Bos indicus* cattle.

The intramuscular and subcutaneous adipose tissues are the most important fat deposits associated with meat quality traits in cattle. The expression level of adipogenic and lipogenic genes in the adipose tissue is regulated by several transcription factors [15, 16]. Fatty acid metabolism is a complex process, which includes lipolysis of dietary fat and its biohydrogenation in the rumen, *de novo* synthesis of FAs by rumen bacteria, absorption and transport of FAs by the host animal, *de novo* synthesis in the host’s tissues, elongation and desaturation in the animal’s tissues, hydrolysis of triglycerides and esterification, and the oxidation of FA or its metabolization into other components [17–21].

Up to date, there are few studies using RNA-seq technique to identify differentially expressed genes (DEG) associated with intramuscular FAs composition in domestic animals. Ramayo-Caldas et al. [22] identified DEG in the liver of crossbred swine (Iberian x Landrace) for groups with extreme values for intramuscular FAs composition. Costa et al. [23] used bulls from different genetic groups of Alentejana and Barrosã breeds with divergent diets, high and low concentration of silage, to identify DEG associated with lipid metabolism in subcutaneous adipose tissue and in the *longissimus lumborum* muscle. Recently, [24] studied the gene expression pattern in taurine cattle finished in different diets with extreme phenotypes for FA profile in the intramuscular fat.

Transcriptomic studies should contribute to elucidate the genetic and non-genetic mechanisms that determine beef FAs composition in the intramuscular fat. These studies could also identify genomic regions and metabolic pathways involved in those mechanisms, aiming to improve the biological knowledge associated with beef FAs composition. Due to the limited number of studies and the implications of intramuscular FAs composition on beef palatability and on human health, it is essential the ongoing study of gene expression for beef FAs composition in Nellore cattle. Moreover, livestock production in Brazil is one of the world’s most important food commerce. In addition, the Brazilian beef production is the second largest in the planet, with 80 % of the herds having the influence of zebu cattle (*Bos indicus*) on its composition [25].

Thus, this study aimed to identify DEG in Nellore cattle finished in feedlot conditions with extreme phenotypes for intramuscular FAs composition in *longissimus thoracis* (LT) muscle by RNA-seq technique.

## Methods

### Animals and information management

Samples were obtained from a total of 48 Nellore young bulls, sons of six sires, belonged to a Capivara farm located in São Paulo state, Brazil, which participates in the Nellore Qualitas breeding program. Animals were selected based on growth, finishing and sexual precocity traits.

Animals were raised on grazing conditions using *Brachiaria* sp. and *Panicum* sp. forages, and free access to mineral salt. After yearling, the breeding animals were selected and the remaining was kept in feedlots for a period of 90 days. The diet was based on whole-plant silage and mix of sorghum grain, soybean meal or sunflower seeds were used as concentrate, with a concentrate/roughage ratio from 50/50 to 70/30.

Animals were slaughtered with an average age of 24 months and 550 kg of liveweight in commercial slaughterhouses, in accordance with the Brazilian Federal Inspection Service procedures. After 48 h post mortem at 0–2 °C, the samples were removed from the *longissimus thoracis* muscle (at least 3.0 kg, including muscle and bone), from between the 12–13th ribs from each animal (left half carcass). Samples were placed in airtight plastic bags and stored at −80 °C for the analyses described below.

### Extraction of lipids

The total lipid concentration was quantified at the Animal Product Technology Laboratory in the Technology Department of FCAV/UNESP according to the method described by [26]. Raw and ground meat samples from *longissimus thoracis* muscle with approximate 3.0 g were transferred into a 250 mL erlenmeyer flask, where 10 mL of chloroform, 20 mL of methanol and 8 mL of distilled water was added. After homogenizing the samples with glass rods, the flasks were placed on a horizontal shaker table (HITACHI High-Speed Micro Centrifuge model CF16RN himac) for 30 min. Later, 10 mL of chloroform and 10 mL of a 1.5 % aqueous sodium sulfate solution were added and the samples were shaken for more two minutes, transferred to 50 mL falcon tubes and then centrifuged at 1,000 × g for two minutes at room temperature. After centrifugation, the supernatant was discarded and the remainder was passed through filter paper to separate the meat fragments from the solution that contained the extracted lipids. The samples were filtered into 25 mL measuring cylinders. The filtrate value was kept to be used in the total lipid calculation and 5 mL was transferred to a 50 mL pre-weighed beaker, oven-dried, cooled in a desiccator for at least 24 h, placed in an oven at 110 °C until complete solvent evaporation, cooled in a desiccator (O/N) and weighed once again. Differences in the initial weight of the beaker (without sample) and final weight (with sample after complete evaporation of solvent) were used to determine the total lipid concentration of samples.

### Fatty acids composition

Fatty acid composition was determined for each sample using the extraction method described by [27]. Muscle samples (~100 g) were collected and grounded for FAs composition. The lipids were extracted by homogenizing the sample with a chloroform and methanol (2:1) solution. NaCl at 1.5 % was added and so that the lipids were isolated.

The isolated lipids were methylated and the methyl esters were formed according to [28]. The FAs composition was quantified using a gas chromatography (GC-2010 Plus - Shimadzu AOC 20i auto-injector) with a SP-2560 capillary column (100 m × 0.25 mm in diameter with 0.02 mm thickness, Supelco, Bellefonte, PA). The initiating temperature was 70 °C with gradual warming (13 °C/min) up to 175 °C, holding for 27 min, and later a further increase of 4 °C/min until 215 °C was reached and held for 31 min. The FAs were identified by comparison of retention time of methyl esters of the samples with standards of C4–C24 (F.A.M.E mix Sigma®), vaccenic acid C18:1 trans-11 (V038-1G, Sigma®) C18:2 trans-10 cis-12 (UC-61 M 100 mg), CLA e C18:2 cis-9, trans-11 (UC- 60 M 100 mg), (Sigma®) and tricosanoic acid (Sigma®). The FAs were quantified by normalizing the area under the curve of methyl esters using *Software* GS solution 2.42. The FAs were expressed in percentage of total FA methyl ester. The FA composition in meat was performed at the Meat Science Laboratory (LCC) in the Department of Animal Nutrition and Production at FMVZ/USP.

Based on the identified acids, 14 FAs (seven individuals and seven groups of FAs) were selected due to their importance in human health. The following FAs were determined: myristic (C14:0), palmitic (C16:0), stearic (C18:0), myristoleic (C14:1), oleic (C18:1 cis-9), linoleic (C18:2 cis9 cis12 n6), conjugated linoleic acid (CLA) C18:2 cis9 trans11, alfa linolenic (C18:3 n3), sum of saturated fatty acid (SFA: C4:0 + C6:0 + C8:0 + C10:0 + C11:0 + C12:0 + C13:0 + C14:0 + C15:0 + C16:0 + C17:0 + C18:0 + C21:0 + C24:0), sum of MUFA (MUFA: C16:1 + C17:1 c10 + C18:1 t11 + C15:1 c10 + C20:1 c11 + C24:1 + C22:1 n9 + C18:1c9 + C14:1 + 18:1 n − 7 + C18:1 n9t), sum of polyunsaturated fatty acid (PUFA: C18:2 cis9 trans 11 + C18:2 trans10 cis12 + C18:2 n6 + C18:3 n3 + C18:3 n6 + C20:3 n3 c11, c14, c17 + C20:3 n6 c8, c11, c14 + C20:4 n6 + C20:5 n3 + C22:6 n3), ratio between PUFA and SFA (PUFA/SFA), sum of ω3 (C18:3 n3 + C20:3 n3 c11, c14, c17 + C22:6 n3 + C20:5 n3), ω6 (C18:3 n6 + C20:3 n6 c8, c11, c14 + C18:2 n6 + C20:4 n6) and ratio between ω6 and ω3.

### RNA-seq quantification

Total RNA was extracted for each sample with TRIzol® reagent (Life Technologies, Carlsbad, CA, USA) from 100 mg of frozen LT muscle. RNA integrity was verified by Agilent 2100 Bioanalyzer® (Agilent, Santa Clara, CA, USA), where only samples with RIN > 8 were used. A total of 2 μg of RNA from each sample was used for library preparation according to the protocol described in TruSeq RNA Sample Preparation kit® v2 guide (Illumina, San Diego, CA, USA). The resultant libraries were quantified using a KAPA Library Quantification kit® (KAPA Biosystems, Foster City, CA, USA), according to Illumina's library quantification protocol. Finally, libraries were pooled (six pools of eight samples each) to perform multiplexing sequencing process, which adds an individual barcode sequences to each sample allowing that each one can be distinguished and analyzed separately during the data analysis. Six lanes of a sequencing flowcell, using the TruSeq PE Cluster kit v3-cBot-HS kit (Illumina, San Diego, CA, USA), were clustering and sequenced using HiSeq (Illumina, San Diego, CA, USA) with a TruSeq SBS v3-HS Kit (200 cycles), according to manufacturer’s instructions. Paired-end reads of 2 × 100 bp were produced. The sequencing analyses were performed at the Genome Center at ESALQ, Piracicaba, São Paulo, Brazil.

### Alignment of sequence reads and transcript assembly

The sequencing data for each sample generated by HiSeq System platform was converted to FastQ format, and separated by libraries (multiplexed data) through Casava software available at https://support.illumina.com/sequencing/sequencing_software/casava.html. The Tuxedo pipeline [29], which includes FastQC (version 0.10.1), TopHat2 (version 2.0.9) and Cuffdiff (version 2.1.1) program were performed in this transcriptomic study using the iPlant Collaborative platform [30]. The FastQC program was used to analyze the sequencing data quality, subsequently, the TopHat2 package was performed to align the reads against the *Bos taurus* virtual transcriptome internally built by TopHat using the UMD3.1 reference genome, containing 24,616 genes. This program was also used to identify the splice junctions of exons transcripts showing the potential exons. For each library, a file was generated with extension ".bam" containing the aligned reads in relation to the reference genome.

Cufflinks (version 2.0.2) was used to assemble the aligned read for each sample individually, providing a parsimonious set of transcripts and to estimate transcript abundances in FPKM (Fragments Per Kilobase of exon per Million fragments mapped) which normalizes transcript expression for transcript length and the total number of sequence reads per sample.

### DEG analysis and functional enrichment

Fatty acid concentration was classified into two extreme phenotype values groups (HIGH and LOW FA concentration) to identify DEG for each FA in 48 samples. Ten animals or biological replicates composed each FA concentration group. Different animals composed those two groups for each beef FA, since the same animal was not necessary extreme for different beef FA. Cuffdiff program included in Tuxedo pipeline performed differential expression analysis. The false discovery rate (FDR) threshold used in this analysis was 10 %. Database for Annotation, Visualization, and Integrated Discovery (DAVID) v6.7 [31, 32] was used for functional enrichment analyses using the list of DEG for each FA and the *Bos taurus* annotation file as background.

## Results

### Phenotypic variation between groups

The descriptive statistics and the analysis of variance for the FA concentration (expressed in % FA) for HIGH and LOW groups are described in Table 1. The coefficient of variation ranged from 0.68 to 10.8 %, indicating a high homogeneity within each group. There were significant differences (*p* < 0.01) between the HIGH and LOW groups for the concentration of all beef FAs measured (Table 1).

**Table 1:** Descriptive statistics and analysis of variance for the fatty acids^a^ for groups of animals with extreme phenotypes (LOW and HIGH)

Fatty acid	Terminology	LOW group^b^				HIGH group^c^				*P*-value
		Min	Max	Mean	SD	Min	Max	Mean	SD	
C14:0	Myristic acid	0.95	1.68	1.38	0.22	2.49	3.73	2.88	0.35	< 0.01
C16:0	Palmitic acid	16.54	20.35	18.84	0.42	23.24	28.57	24.48	0.42	
C18:0	Stearic acid	10.86	12.80	11.80	0.22	15.88	17.78	16.68	0.22	
C18:1 cis-9	Oleic acid	25.57	29.51	27.60	0.38	34.51	37.51	35.85	0.38	
C18:2 cis-9 trans-11	CLA	0.14	0.20	0.18	0.02	0.33	0.59	0.41	0.02	
C18:2 cis-9 cis −12	Linoleic acid	2.47	4.92	4.13	0.28	8.96	11.82	9.98	0.27	
C18:3 n3	α-Linolenic acid	0.23	0.49	0.41	0.03	0.87	1.20	1.00	0.03	
SFA	Sum of SFA	39.91	42.05	41.10	0.32	45.37	49.62	46.87	0.32	< 0.01
MUFA	Sum of MUFA	30.14	34.50	32.75	0.39	40.44	43.71	42.20	0.39	
PUFA	Sum of PUFA	4.33	8.24	7.16	0.46	15.79	20.46	17.21	0.46	
PUFA/SFA	PUFA and SFA ratio	0.09	0.18	0.16	0.01	0.36	0.51	0.40	0.01	
n3	Sum of n-3	2.78	5.39	4.49	0.29	9.77	12.94	10.89	0.29	< 0.01
n6	Sum of n-6	1.24	2.65	2.18	0.20	5.14	7.62	6.14	0.20	
n6/n3	n6 and n3 ratio	1.45	1.64	1.57	0.03	2.15	2.63	2.27	0.03	

^a^The concentration of fatty acids are expressed as a percentage of total fatty acid methyl esters (FAME); ^b^LOW group: ten lowest extreme phenotypes; ^c^HIGH group: ten highest extreme phenotypes

### Throughput sequencing, read mapping and assembly

The Table 2 presents the sequencing throughput and mapping statistics for each HIGH and LOW groups. The sequence quality was assessed through the distribution of transcript abundance for each FA and gene expressed as a box-plot of the log of fragments per kilobase of exon per million fragments mapped (FPKM) values (Additional file 1). For each FA, similar median and quartiles values for FPKM estimates were obtained between the HIGH and LOW groups.

**Table 2:** Average number of pair-end reads, number of mapped reads and concordant pair alignment rate (%) for HIGH and LOW^a^ groups for each beef fatty acid

Fatty acid	HIGH group^a^			LOW group^b^		
	Input reads	Mapped reads	Concordant pair alignment rate (%)	Input reads	Mapped reads	Concordant pair alignment rate (%)
SFA	20071041	14437527	72.4	19638545	14820629	74.5
MUFA	20240465	14684305	72.8	16939278	12297985	72.4
PUFA	17185305	12864023	73.6	20237062	14549977	72.4
PUFA/SFA	16414850	12317003	73.7	20237100	14568890	72.5
n3	17139100	12827764	73.6	20222528	14551467	72.4
n6	17185305	12864023	73.6	20847167	15061920	72.7
n6/n3	16210236	11638824	72.4	16917230	12764008	74.1
C14:0	18018166	12949199	72.4	17468012	13076106	73.6
C16:0	19244978	13835678	72.3	17828062	13334095	73.6
C18:0	18384047	13412632	73.1	17381770	13032452	73.9
C18:1 cis-9	19653791	14182228	72.4	15553754	11278425	72.4
C18:2 cis-9 trans-11	20179794	15010030	74.1	18123403	13240952	73.1
C18:2 cis-9 cis −12	17185305	12864023	73.6	20847167	15061920	72.7
C18:3 n3	18224781	13583864	73.4	19220721	13826217	72.4

^a^HIGH group: Ten highest extreme phenotypes; ^b^LOW group: Ten lowest extreme phenotypes

The principal component analyses of FPKM values for all genes indicated that there were sufficient number of DEG to differentiate HIGH and LOW groups for most of the FAs (Additional file 2).

Moreover, the expression profiles of selected housekeeping genes were evaluated, such as the Hypoxanthine phosphoribosyltransferase 1 (*HPRT1*) and Tyrosine 3-Monooxygenase /Tryptophan 5-Monooxygenase activation protein, Zeta (*YWAZ*). For both genes, the expression patterns were similar between HIGH and LOW groups for all beef FAs evaluated.

### Differential Expression Genes (DEG)

Differential expression analysis between the HIGH and LOW groups identified 13 DEG for C14:0, 35 for C16:0, 187 for C18:0, 371 for C18:1 cis-9, 24 for the C18:2 cis-9 trans-11 (CLA), 89 for C18:2 cis-6 cis −12 n6 and 110 for C18:3 n3 FA. For the respective sums of the individual FAs, 51 DEG for SFA, 336 for MUFA, 131 for PUFA, 55 for ω3, and 627 for ω6 were identified. For PUFA/SFA and ω6/ ω3 ratio, 92 and 22 DEG were identified, respectively. The list of the DEG identified between groups with different FAs composition is described in Table 3. (Additional file 3).

**Table 3:** Description of differentially expressed genes identified between groups of Nellore bulls with different fatty acid composition in *longissimus thoracis* muscle

Ensembl_gene_ID	Gene symbol	BTA: locus	Function	FA	Fold change	*q* value
ENSBTAG00000033803	*FABP7*	9:28834077–28837863	Cytosolic fatty-acid and lipid binding	C18:3 n3	27.891	0.010
ENSBTAG00000022570	*LOC782922*	13:43947620–44116989	Fatty acid metabolic process	MUFA	−2.100	0.007
ENSBTAG00000004860	*SLC27A6*	7:26237928–26329594	Fatty acid transporter	MUFA	0.855	0.004
ENSBTAG00000012885	*ACAT1*	15:17999931–18028984	Fatty acid metabolism, Synthesis and degradation of ketone bodies	n6	0.444	0.047
ENSBTAG00000005105	*PAFAH2*	2:127684836–127720396	Lipid catabolic process	C18:0	−0.750	0.050
ENSBTAG00000022449	*SCD5_BOVIN*	6:99233278–99410753	Fatty acid metabolic process; fatty acid, unsaturated fatty acid and lipid biosynthetic process	C18:0	0.821	0.030
ENSBTAG00000001444	*TNXB*	23:27083668–27136954	Fatty acid metabolism	C18:1cis-9	−0.640	0.004
ENSBTAG00000006716	*PTGS1*	11:93219286–93245045	Fatty acid metabolic process; fatty acid and unsaturated fatty acid biosynthetic process	C18:1cis-9	−0.824	0.010
ENSBTAG00000007763	*SLC1A4*	11:63290421–63395507	Carboxylic acid transport	C18:0	−1.502	0.006
ENSBTAG00000015228	*CD74*	7:63748884–63756646	Organic acid and lipid biosynthetic process	C18:0	0.605	0.033
ENSBTAG00000016819	*FABP3*	2:122723224–122783830	Cytosolic fatty-acid and lipid binding	PUFA/SFA	0.895	0.011
ENSBTAG00000011917	*GPAM*	26:32963413–33003349	Fatty acid metabolic process	PUFA/SFA	−0.664	0.011
ENSBTAG00000018248	*MGLL*	22:60443563–60493810	Lipid metabolism	PUFA/SFA	−0.620	0.011
ENSBTAG00000038321	*LIPE*	18:51216018–51227395	Lipid catabolic process, and Insulin signaling pathway	PUFA/SFA	−0.620	0.011
ENSBTAG00000004178	*ACOX2*	22:43379503–43410315	Metabolic and catabolic process, beta-oxidation of fatty acid, lipid catabolic process and organic acid catabolic process	n6	−0.486	0.025
ENSBTAG00000008063	*PPARA*	5:117151548–117233112	Fatty acid metabolic process	n6	−0.534	0.003
ENSBTAG00000017542	*PPARD*	23:9340954–9353750	metabolic and catabolic process, beta-oxitation, transport and oxidation of fatty acids	n6	−0.643	0.011
ENSBTAG00000033089	*PTPLA*	13:32347675–32369621	Biosynthesis of unsaturated fatty acids	ω6	0.500	0.016
ENSBTAG00000007331	*PLOD2*	1:123322466–123444003	Carboxylic acid binding	C18:2cis-9cis-12 PUFA	0.886 0.975	0.013 0.008
ENSBTAG00000001417	*ACSM1*	25:18349701–18413889	Metabolic and biosynthetic process of fatty acid; biosynthetic process of lipid, organic acid and carboxylic acid.	n3 n6	−176.413 −132.855	0.013 0.006
ENSBTAG00000019813	*ADIPOQ*	1:81005167–81018328	Metabolic and catabolic process, beta-oxidation and oxidation and regulation of metabolic of fatty acid	n3 n6	−118.366 −0.900	0.013 0.005
ENSBTAG00000037526	*FABP4*	14:46833664–46838053	Cytosolic fatty-acid binding	n3 ω6	−135.344 −0.927	0.013 0.016
ENSBTAG00000001154	*DGAT2*	15:55940756–55973229	Lipid biosynthetic process	C18:2cis-9cis-12 C16:0 PUFA/SFA	−0.626 0.654 −0.960	0.025 0.031 0.011
ENSBTAG00000021287	*SLC16A7*	5:53987908–54214799	Monocarboxylic acid transmembrane transporter activity	C14:0 C18:0 n3	0.816 0.860 −167.220	0.040 0.018 0.013
ENSBTAG00000005259	*UCP3*	15:54213565–54224051	Fatty acid metabolic process	C18:1cis-9 MUFA n6	0.615 0.855 −0.735	0.023 0.004 0.002
ENSBTAG00000016864	*LBP*	13:67874473–67910095	Lipid transport	C14:0 C16:0 SFA	1.950 1.880 186.970	0.040 0.006 0.014
ENSBTAG00000012855	*LPL*	8:67481088–67511227	Metabolic and biosynthetic process of fatty acid	C16:0 C18:1cis-9 PUFA/SFA n3	0.870 −0.568 −114.732 −0.841	0.02 0.033 0.011 0.042
ENSBTAG00000015978	*ANXA1*	8:49624472–49642916	Transport of lipid, monocarboxylic acid, organic acid, fatty acid, long-chain fatty acid and carboxylic acid; arachidonic acid secretion	C18:0 C18:1cis-9 MUFA PUFA n6	0.826 −0.891 −0.740 0.717 0.646	0.006 0.004 0.004 0.008 0.002
ENSBTAG00000006447	*ACSM3*	25:18605634–18656582	Lipid metabolism	C18:2cis-9cis-12 MUFA PUFA SFA n3	−1.853 2.455 −1.965 −19.001 −173.510	0.012 0.004 0.008 0.033 0.023
ENSBTAG00000004281	*ACSS1*	13:42963403–43076853	Acetyl-CoA metabolic process	C14:0 C16:0 C18:0 C18:1cis-9 SFA n3	1.184 1.088 1.531 −1.180 122.248 −147.662	0.040 0.043 0.006 0.032 0.033 0.013
ENSBTAG00000016514	*CPE*	17:546397–697915	Insulin processing	C14:0 C16:0 C18:1cis-9 SFA MUFA n3	1.490 1.683 −1.399 139.560 −1.279 −101.4	0.040 0.006 0.038 0.014 0.004 0.049
ENSBTAG00000000448	*BDH1*	1:72572940–72608810	Synthesis and degradation of ketone bodies	C14:0 C16:0 C18:0 SFA C18:2cis-9cis-12 MUFA n3	1.045 1.212 1.534 105.874 −0.854 −0.925 −174.448	0.040 0.020 0.006 0.014 0.043 0.004 0.013

**Fig. 1:**
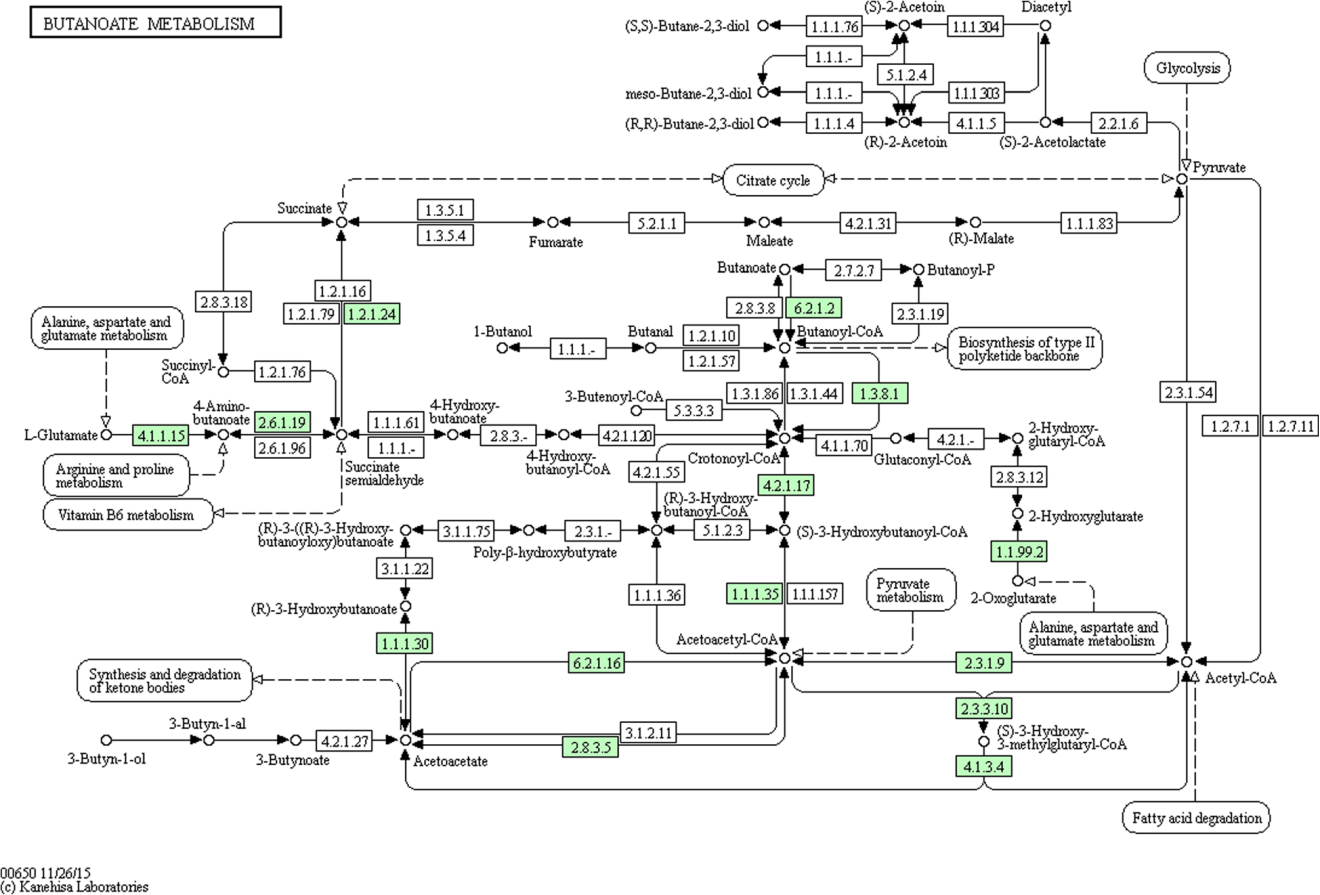
Metabolic pathways of ACSM3 gene (6.2.1.2) [70]

**Fig. 2:**
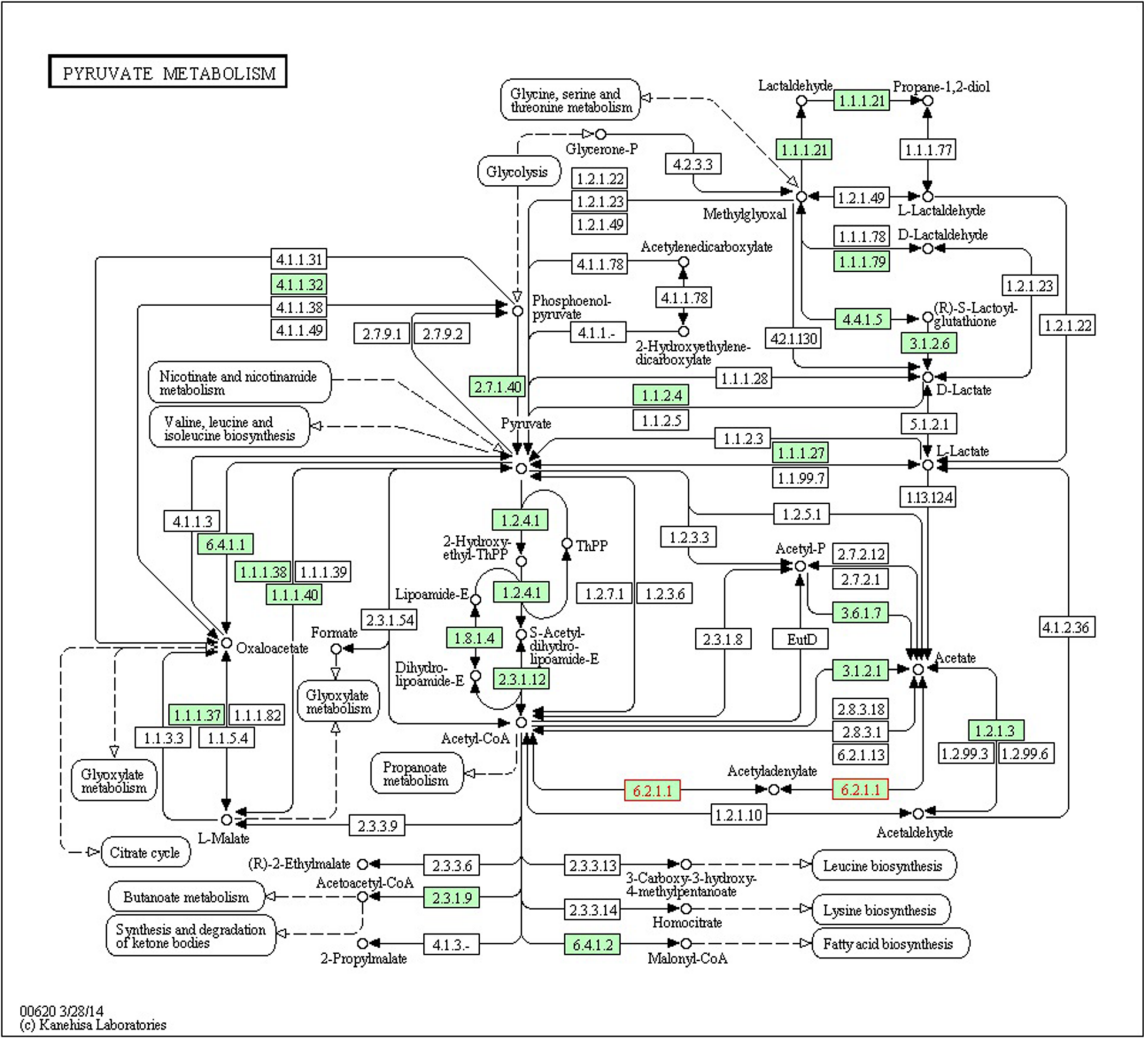
Metabolic Pathways of ACSS1 gene (6.2.1.1) [71]

The *ACSM3* (acyl-CoA synthetase medium-chain family member 3) gene, that was differentially expressed for linoleic, MUFA, PUFA, SFA and ω3 acids, participates in the metabolism of lipids and in metabolic pathways that involves the precursor acetyl-CoA metabolism (Fig. 1). Following the process of FAs synthesis, the *ACSS1* (acyl-CoA synthetase short-chain family member 1) gene acts in the transformation of acetyl-CoA into FAs through chemical reactions and metabolic pathways involving acetyl-CoA (Fig. 2). This gene was differentially expressed (q <0.05), upregulated for SFA such as palmitic, stearic, oleic and SFA sum, and downregulated for unsaturated acids, such as ω3.

Other important DEG (*q* < 0.05) identified was the *SLC16A7* (solute carrier family 16 (monocarboxylate transporter), member 7), which was upregulated for SFA sum, myristic and stearic acids, and downregulated for ω3. This gene is responsible for catalyzing the transfer of monocarboxylic acids from one cell to another. Other genes such as *ANXA1* (annexin A1), upregulated for stearic acids, ω6 and PUFA sum, and downregulated for oleic and MUFA; and the *LBP* (lipopolysaccharide binding protein) gene upregulated for the myristic, palmitic and SFA sum, are also responsible for the transport of FAs between cells through pores of a carrier or agent.

The SLC27A6 (solute carrier family 27 (FA transporter), member 6) gene was upregulated for SFA and acts as a carrier of FAs. The *ACSM1* gene was downregulated for ω3 and ω6, and it participates directly in FAs synthesis. This gene is responsible for the chemical reactions and metabolic pathways that involve FAs and aliphatic monocarboxylic acids of open chain that are naturally released by hydrolysis of the fats and oils.

The *GPAM* (glycerol-3-phosphate acyltransferase, mitochondrial) and *UCP3* (mitochondrial uncoupling protein 3) genes have similar functions, in which the first one was found upregulated for oleic acid and MUFA and downregulated for ω6, while the second gene was downregulated for PUFA/SFA ratio. The stearoyl-CoA desaturase is an integral membrane protein of the endoplasmic reticulum, which catalyzes the synthesis of MUFA from SFA, which can also be a key regulator of energy metabolism.

The *ADIPOQ* (adiponectin, C1Q and collagen domain containing), *PLOD2* (procollagen-lysine, 2-oxoglutarate 5-dioxygenase 2), and *LPL* (lipoprotein lipase) genes were differentially expressed for several FAs, and participate in several processes related to FAs synthesis. In this sense, the *ADIPOQ* gene was downregulated for ω3 and ω6. This gene participates directly in the metabolic pathways related to FAs production, such lipids and organic acids and is also involved in the regulation of cellular ketone metabolic process (lipids and FAs) and in FAs oxidation and beta-oxidation. The *PLOD2* gene acts in the binding of carboxylic acids and in other organic acid containing one or more carboxyl group (−COOH) or anions (COO-) and was upregulated for linolenic FAs and PUFA sum, indicating that its expression may promote the synthesis of PUFA. Finally, the *LPL* gene was differentially expressed for palmitic and oleic acids, ω3 and PUFA/SFA ratio. There was downregulated expression of all unsaturated FAs, indicating that high expression of this gene is associated with a low concentration of these acids in the samples analyzed.

The LOC782922 (prostaglandin F synthetase II-like) gene was downregulated for MUFA, which can act in the metabolism of prostaglandins and participates in the chemical reactions and metabolic pathways of unsaturated FAs synthesis or other FAs containing one or more double bonds between the carbon atoms. The CPE (carboxypeptidase E) gene is responsible for insulin synthesis through proteolysis of its precursor (preproinsulin), which was upregulated for C14:0, C16:0, C18:0 and SFA sum, and downregulated for C18:1 cis-9, MUFA and ω3. While the BDH1 (3-hydroxybutyrate dehydrogenase, type 1) gene was differentially expressed (*q* < 0.05) for C14:0, C16:0, C18:0, ω3, MUFA and SFA sums.

### Functional analysis

Gene ontology (GO) and pathway enrichment analysis were perfomed to gain insight into the predicted gene network. The most significant GO terms were focused on cellular components, molecular functions and biological processes (Table 4). Molecular functions controlling FAs metabolism are highly interconnected and linked with related pathways, such as lipid, carbohydrate metabolism and energy homeostasis pathway. The essential metabolic network for homeostatic control and organism development is constituted by these pathways and its interactions [33]. In this study, molecular functions related to recognize (bind) glycosaminoglycan, polysaccharide and carbohydrate molecules were identified (Table 4).

**Table 4:** Gene Ontology (GO) terms enriched with differentially expressed genes (FDR < 0.1)

GO terms	Number^a^	*P*	FDR
*Cellular components*			
GO:0005576 - extracellular region	106	5,93E-15	7.93E-12
GO:0044421 - extracellular region part	65	8,15E-14	1,10E-10
GO:0031012 - extracellular matrix	39	2,68E-13	3,61E-10
GO:0005578 -proteinaceous extracellular matrix	34	4,96E-11	6,68E-08
*Molecular functions*			
GO:0005539 - glycosaminoglycan binding	22	1,31E-13	1,93E-10
GO:0001871- pattern binding	22	1,03E-11	1,51E-08
GO:0030247- polysaccharide binding	22	1,03E-11	1,51E-08
GO:0030246 - carbohydrate binding	29	1,37E-09	2,01E-06
GO:0008201- heparin binding	13	1,64E-07	2,41E-04
GO:0030528 - transcription regulator activity	64	1,19E-05	0,017513
GO:0003700 - transcription factor activity	44	6,64E-05	0,097714
*Biological process*			
GO:0001501 - skeletal system development	24	6,72E-08	1,16E-04
GO:0030198 - extracellular matrix organization	16	1,81E-07	3,11E-04
GO:0043062 - extracellular structure organization	17	1,06E-06	0,001821
GO:0009611 - response to wounding	26	5,79E-06	0,009976
GO:0006954 - inflammatory response	18	3,19E-05	0,05502
GO:0007517 - muscle organ development	16	4,26E-05	0,073391
GO:0043009 - chordate embryonic development	23	4,66E-05	0,080367
GO:0060537 - muscle tissue development	14	5,11E-05	0,088045
GO:0009792 - embryonic development ending in birth or egg hatching	23	5,13E-05	0,088388

^a^number of differentially expressed genes

The biologicals processes identified are related mainly with extracellular structure and organization, response to wounding, inflammatory response, embryonic development, skeletal and muscle developments (Table 4). Four KEGG (Kyoto Encyclopedia of Genes and Genomes) pathways were identified over represented for DEG by DAVID tool. These pathways were related with ECM-receptor interaction (*P* = 6,90E-7), focal adhesion (*P* = 1,08E-06), PPAR signaling pathway (*P* = 1,85E-05), and TGF-beta signaling pathway (*P* = 0.0049).

## Discussion

### Phenotypic variation between groups

Evaluating *longissimus* muscle of *Bos indicus*, [34] observed similar concentrations of SFA as described in this study, and different for the PUFA, MUFA, PUFA/SFA, ω3, ω6 and ω6/ω3 ratio. Cesar et al. [35] identified genomic regions associated with FAs composition and fat deposition in Nellore steers and found concentrations of FAs that are close to the average obtained for the HIGH groups in the present study.

### Differential Expression Genes (DEG)

In ruminants, the FAs synthesis occurs mainly in the adipose tissue, except during the lactation, when the mammary gland becomes the predominant organ [36]. The main point about FAs synthesis control is the acetyl-CoA carboxylase, and it seems that the endocrine control is very similar in, at least, adipose tissue (insulin activation, inhibition of catecholamine) of ruminants and non-ruminants [37]. Acetate is the principal precursor of FAs synthesis in ruminants, and must be converted to acetyl-CoA by the action of acetyl-CoA synthetase and then incorporated into FAs. The adipose tissue is largely responsible for the conversion of acetate into acetyl-CoA, and consequently, the greatest synthesizer of FAs in ruminants [38].

From the results obtained in this study, it was possible to highlight some important genes related to biologicals processes involved in beef’s FAs synthesis, such as those involved in the transport of essential components in animal tissues. The myristic and palmitic FAs are considered to be hypercholesterolemic, and are responsible for increasing the amount of low density lipoproteins (LDL), which expand the risk of heart diseases [39]. Other genes that also operate in the transport of FAs were identified, but the DEG were only for a single FA, such as *FABP7* (FA binding protein 7, brain) and *FABP3* (FA binding protein 3, muscle and heart) genes, which appeared upregulated for linolenic acid and PUFA/SFA ratio, respectively. These genes produce proteins that apparently play a role in intracellular transport of long chain FAs and their acyl-CoA esters. The intracellular FAs binding proteins (FABPs) belong to a multigene family. The FABPs are divided into at least three distinct groups: hepatic, intestinal, and cardiac. These form 14–15 kDa protein and participate in the absorption, metabolism and/or intracellular transport of long chain FAs, and may also be responsible for growth modulation and cell proliferation (provided by RefSeq, July 2008). Regarding the *FABP4* (FA binding protein 4, adipocyte) gene, it works in FAs binding proteins and it was downregulated for ω3 and ω6. The FABPs are often associated with lipid metabolism by acting as intracellular transport of hydrophobic intermediates and lipids metabolites trough the membranes. The *PAFAH2* gene, downregulated for oleic acid, participates in chemical reactions and pathways that break lipids. The *MGLL* (monoglyceride lipase) gene, downregulated for PUFA/SFA ratio, operates in the chemical reactions for lipids synthesis and acts as a catalyst in FAs synthesis reactions.

**Fig. 3:**
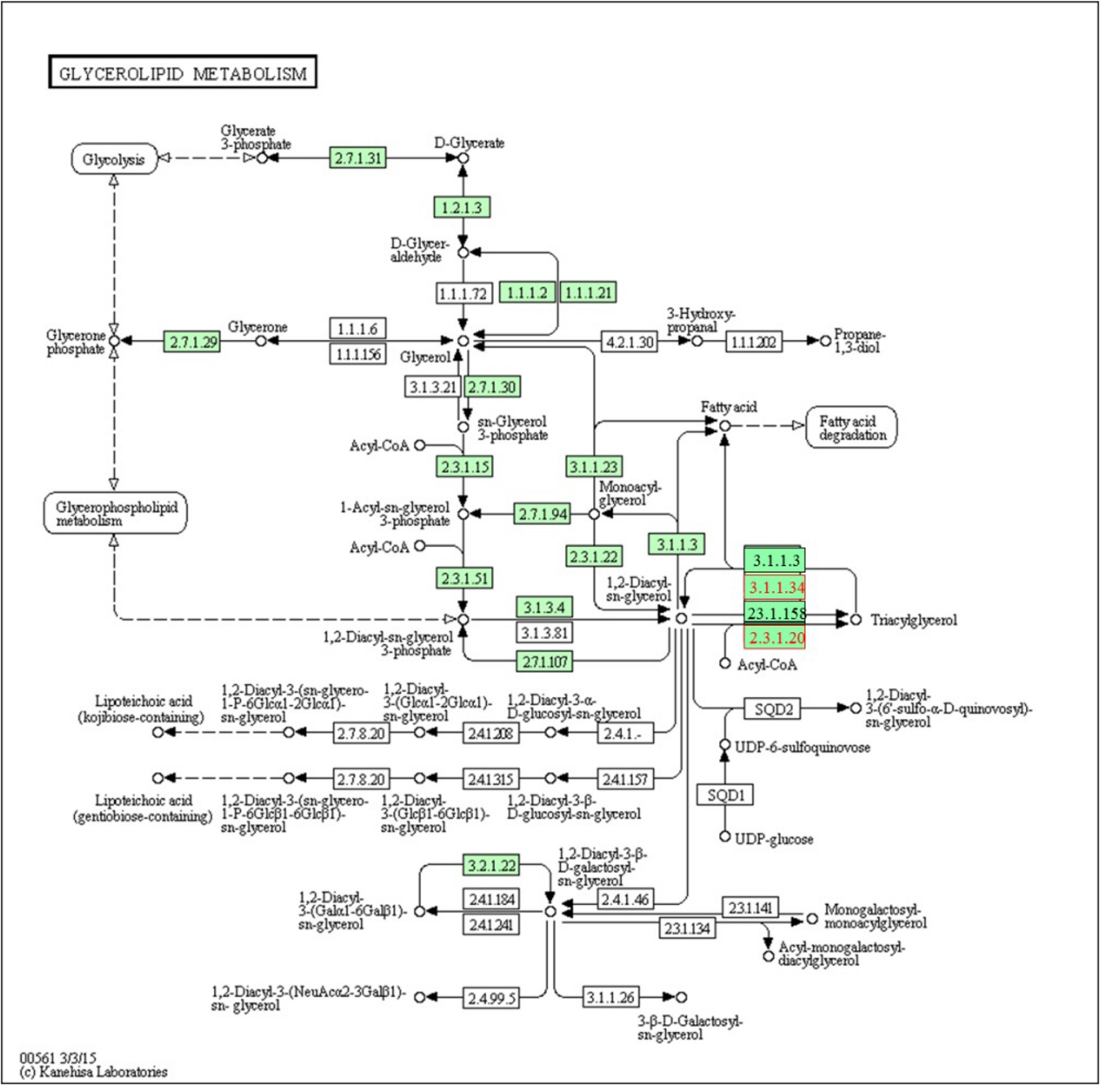
Metabolic pathway of DGAT2 (2.3.1.20) and LPL (3.1.1.34) genes [72]

Our results showed some genes directly associated with FAs synthesis. In this sense, the *DGAT2* (diacylglycerol O-acyltransferase 2) gene, upregulated for palmitic and downregulated for linoleic acid and PUFA/SFA ratio, is essential for the triglycerides synthesis and intracellular storage [40] found negative correlations between marbling and concentrations of stearic, linoleic acid, and PUFA [24] reported a positive and moderate correlation between the level of marbling and the expression of *DGAT2* gene. The *DGAT2* gene is an important contributor to the triacylglycerol synthesis through their acyltransferase activity. As the amount of triglyceride within the adipocyte increases, the total proportion of SFA also increases in relation to other ones [41]. An increase in *DGAT2* gene expression was previously demonstrated to be associated with an increase in the amount of intramuscular fat [42]. Thus, these results demonstrate that *DGAT2* gene contributed to the accumulation of SFA in the intramuscular tissue during the finishing phase (Fig. 3).

The *LPL* gene plays a role in chemical reactions and metabolic pathways that result in FAs synthesis and open-chain monocarboxylic acids, which can be released by hydrolysis that occur in fats and oils. The activity of this gene in the adipose tissue and the subsequent increase in deposition of triglycerides are promoted by insulin [43]. Some studies have suggested that the FAs synthesis in the subcutaneous adipose tissue on beef is not sensitive to insulin levels [44, 45]. In this sense, the activity of LPL gene in the muscle tissue appears not to be insulin dependent [43], however, many authors have provided evidence to support the opposite [46, 47].

**Fig. 4:**
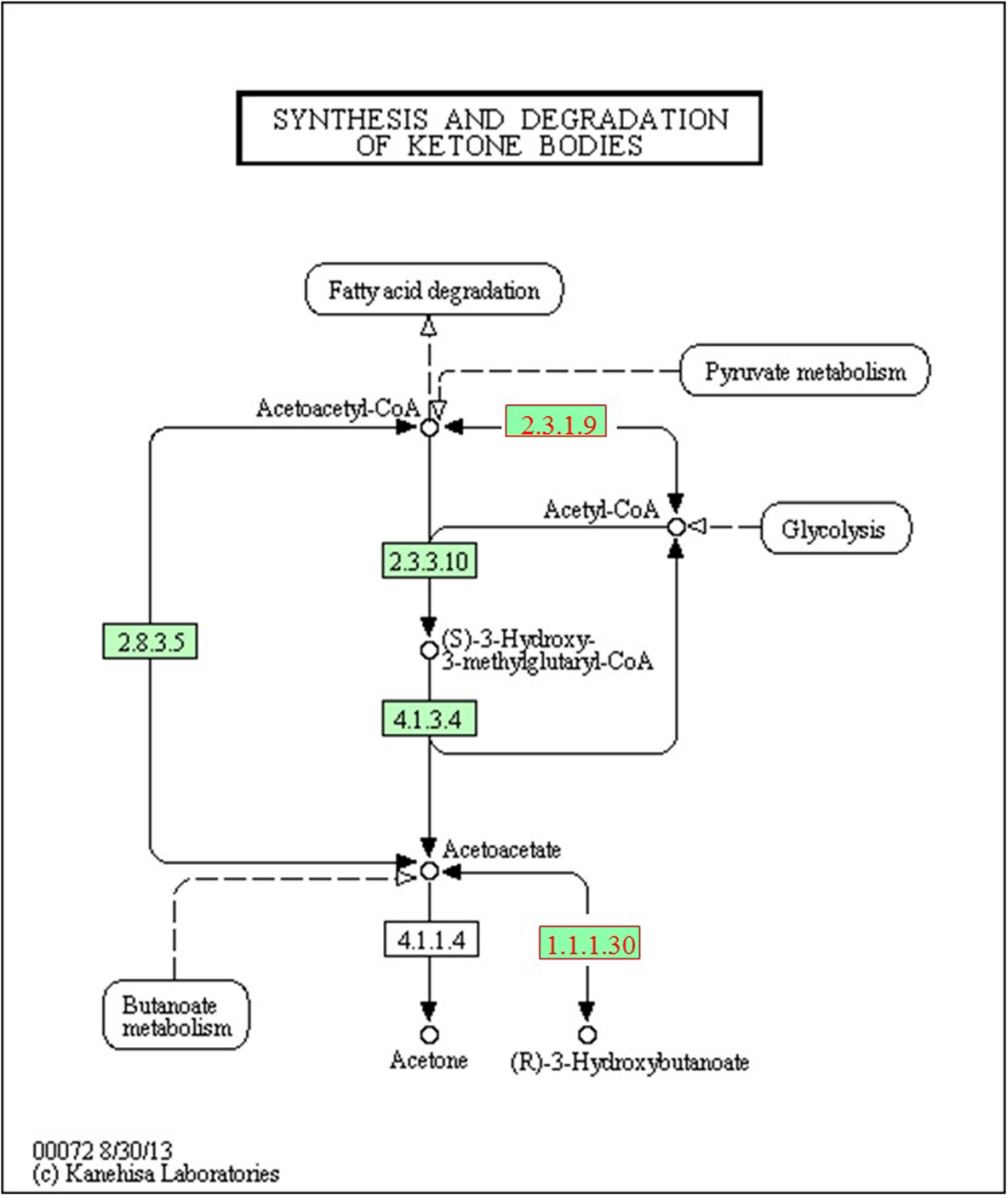
Metabolic pathway for BDH1 (1.1.1.30) and ACAT1 (2.3.1.9) genes [73]

The BDH1 gene was upregulated for SFA and downregulated for unsaturated FAs, indicating a higher gene expression as the degree of saturation of the sample is increased. This gene is responsible for the synthesis and degradation of ketone bodies, which allows transporting the energy obtained by the oxidation of FAs to the peripheral tissues, then to be used in the ATP synthesis in the absence of carbohydrates in the diet (Fig. 4). This fact justifies the greatest expression of this gene in the presence of SFA for the synthesis of ketone bodies from SFA, since it is less complex when compared to the degradation of unsaturated FAs. The *ACAT1* (acetyl-CoA acetyltransferase 1) gene has the same function of *BDH1* gene, and was upregulated for ω6 (Fig. 4).

The results showed an evident antagonism for the expression of some DEG related to FAs synthesis. In this sense, some of those genes that were upregulated for SFA group were also downregulated for MUFA and PUFA groups. Animals with high degree of fatness or deposition of intramuscular fat in LT muscle presented higher concentrations of SFA and less concentrations of PUFA. De Smet et al. [48] and Wood et al. [3] showed that when the proportion of animal fat increases, the proportion of PUFA in meat decreases drastically.

Ruminants incorporate essential FAs, especially phospholipids, in muscle lipids rather than storing them in fat [3]. Recently, more emphasis has been dedicated on muscle due to its importance as a protein source and as a growing aversion to visible fat at retail. The muscle also contains higher concentrations of ω6 and ω3 acids, whose importance to human nutrition have been recently recognized. In recent years, the procedures for separation and identification of low levels of unsaturated FAs in muscle have been greatly improved [3].

Thus, the development of molecular genetics, particularly high throughput sequencing methods, provides a unique opportunity to identify genes and pathways associated with diseases and complex traits [49]. However, recent studies revealed a limitation of genome-wide association study (GWAS) to identify loci with significant effects on different populations of the same breed, since many discordant genomic regions have been identified [50]. Tizioto et al. [51] observed DEG using RNA-seq method and also applied GWAS to identify genomic regions for feed efficiency in Nellore cattle. These authors found several biological mechanisms and attributed the differences in the candidate regions/genes to the specific modulation of mRNA.

In a recent GWAS study, [52] used a population with 963 Nellore bulls, which contained 48 animals used in this study, and identified several genomic regions which explained more than 1 % of the additive genetic variance for beef FAs composition. The authors reported some regions near to DEG identified in this study. In this regard, the *ACSM3* and *ACSM1* genes were differentially expressed for ω3 acids and were located in a region (BTA25, 12 Mb) which explained more than 1 % of the additive variance genetic for ω3, as reported by [52]. Those authors also reported QTL detected by GWAS in the same region that the TNXB (tenascin XB) and PPARD (peroxisome proliferator-activated receptor delta) genes were differentially expressed in this study. These genes are related with FAs metabolism, transport and oxidation. These results showed some degree of equivalence, since it was identified common regions between the results of structural analysis (GWAS) and functional analysis (RNA-seq) for beef FAs composition.

The results obtained in this study indicated that beef’s FAs composition in Nellore cattle is influenced by many genes and complex metabolic pathways. Furthermore, it identified genes that contribute to FAs metabolism, through intra or extra-cellular transport of FAs synthesis precursors in intramuscular fat of *longissimus* muscle. Among those genes, some of them must be highlighted, such as: *ACSM3* and *ACSS1* genes, since they work in the FAs precursor synthesis and their subsequent transformation into FAs, respectively. In addition, the *DGAT2* gene that assists the deposition of saturated fat tissue; *GPP* and *LPL* genes that support the insulin synthesis, which stimulates the glucose synthesis as the amino acids entry into the cells; and the *BDH1* gene which is responsible for the synthesis and degradation of ketone bodies, used in the ATP synthesis.

### Functional analysis

Several GDE related to biological processes associated with inflammatory response were identified in the present study. In these sense, stated that the fatty acids influenced inflammatory response acting via cell surface and intracellular receptors/sensors that control gene expression patterns and inflammatory cell signaling. Some effects of FAs on inflammatory cells appear to be mediated or associated with changes in FAs composition of cell membranes [53].

Extracellular matrix (ECM) consists of a complex of structural and functional macromolecules with an important role in tissue, organ morphogenesis, cell maintenance, and tissue structure and function. It can directly or indirectly influence specific cellular activities such as cell adhesion, proliferation, differentiation, and migration [54, 55]. In muscle tissue, cells are tightly bound together, and the extracellular spaces containing the extracellular matrix are limited. These results corroborates with previous study of transcriptome profile of Nellore steers with different genomic breeding value of intramuscular fat deposition [56]. Jiang et al. [55] studying transcriptome comparison between porcine subcutaneous and intramuscular stromal vascular cells during adipogenic differentiation speculated that the ECM-receptor interaction pathway might participate in intramuscular stromal vascular cell differentiation process. Lee et al. [57] studied the difference of the depot specific gene expression from different adipose tissues of omental, subcutaneous and intramuscular tissues in cattle, and identified the ECM-receptor interaction with the one of commonly enriched pathways in all adipose tissues and also functioned as a sub-pathway of other enriched pathways. These authors suggested that the interactions between ECM components and transmembrane receptors of fat cells depend on the depot specific adipogenesis.

The most overexpressed genes identified in this study related to muscle and skeletal developments could be good candidates for Nellore breeding programs in which the main goal is to enhance meat and carcass quality. Studying a Hanwoo beef cattle population, [58] identified pathways related to cell adhesion regulation, structure, integrity, and chemokine signaling pathway upregulated in intramuscular adipose but downregulated in the muscle. Cui et al. [54] also proposed that these pathways play an important role in the intramuscular fat deposition in chicken. Cánovas et al. [59] identified the ECM-receptor interaction and *TGF-beta* signaling pathways as the most relevant metabolic pathways represented in the list of DEG related with meat composition in pig *longissimus dorsi* muscle.

The transforming growth factor *TGF-beta* signaling pathway is involved in many cellular processes including cell growth, differentiation and apoptosis, cellular homeostasis in both the adult organism and in the developing embryo. Mehla et al. [60] identified the *TGF-beta* signaling pathway related to DEG genes in Zebu cattle due to heat stress effects. Peroxisome proliferator-activated receptors (*PPARs*) are nuclear hormone receptors that are activated by FAs and their derivatives, and play an essential physiological role in the regulation of adipocyte tissue development lipogenesis and skeletal muscle lipid metabolism [61–64]. There are three members of the PPAR family (*PPARalpha*, *beta/delta*, and *gamma*) with different expression patterns in vertebrates. *PPAR alpha* plays a role in lipid metabolism in the liver and in the skeletal muscle, and in the modulation of the inflammatory response. *PPAR beta*/*delta* is involved in lipid oxidation and cell proliferation, and acts on embryo implantation, cell proliferation and apoptosis. *PPAR gamma* is related to cell cycle withdrawal and promotes myocyte/adipocyte differentiation to enhance blood glucose uptake [61, 62, 64–66].

Doran et al. [67] studying GWAS in Holstein-Friesan cattle identified the PPAR signaling pathway as the most significantly overrepresented biological pathway involved in carcass trait performance, suggesting that *PPAR* would also play a key role in controlling carcass weight, carcass fat and carcass conformation traits. He et al. [68] identified an association between genes and SNPs in the *PPAR* signaling pathway with porcine meat quality traits.

Carcass and meat traits, especially those obtained through beef FAs composition of intramuscular fat analyses, are not used by the industry as a criterion for determining the animal’s value for slaughter. However, there is a growing trend in the international meat market to provide technical and scientific guarantees to certify food safety, product quality and its benefits to human health. Therefore, the production of the required information is essential and needed to improve the marketing of beef products. There are few studies about transcriptome in Zebu animals, in which [51] is the unprecedented as it is the first study of gene expression for beef feed efficiency in *Bos indicus* animals. Thus, it provides subsidies to improve the beef quality of Zebu cattle under tropical conditions, producing a healthier food for consumers.

## Conclusion

Several genes related to lipids metabolism and beef FAs composition were found in this study. The identification of such candidate genes must contribute to the elucidation of the genetic basis that determines the beef FAs composition of intramuscular fat in Nellore cattle. This information would contribute to the improvement of meat quality through selection processes, since the molecular processes that control FAs composition and metabolism are not completely understood yet. Moreover, the DEG identified can be used in future studies of fine mapping whose primary function is to search for functional mutations and can be useful to identify some specific variants.

## Additional files

Additional file 1: Boxplot of the transcript abundance distribution for each beef fatty acid and gene expressed. (PDF 42 kb)
Additional file 2: Principal Component Analysis (PCA) of gene FPKM values between the high and low groups with fatty acids profile: (a) PCA for sum of SFA; (b) PCA for sum of MUFA; (c) PCA for sum of PUFA; (d) PCA for sum of PUFA/SFA ratio; (e) PCA for sum of n3;.(f) PCA for sum of n6; (g) PCA for n6/n3 ratio;(h) PCA for myristic acid (C14:0); (i) PCA for palmitic acid (C16:0); (j) PCA for stearic acid (C18:0);(k) PCA for oleic acid (C18:1 cis-9); (l) PCA for conjugated linoleic acid (C18:2 cis-9 trans-11); (m) PCA for linoleic acid (C18:2 cis6 cis12 n6); (n) PCA for α-linolenic (C18:3 n3). (PDF 459 kb)
Additional file 3: Table S1. Differentially expressed genes for C14:0. **Table S2**. Differentially expressed genes for C16:0. **Table S3**. Differentially expressed genes for C18:0. **Table S4**. Differentially expressed genes for C18:1 cis-9. **Table S5**.Differentially expressed genes for C18:2 cis-9trans-11. **Table S6**. Differentially expressed genes for C18:2 cis-6 cis -12 n6. **Table S7**. Differentially expressed genes for C18:3 n3. **Table S8**. Differentially expressed genes for SFA. **Table S9**. Differentially expressed genes for MUFA. **Table S10**. Differentially expressed genes for PUFA. **Table S11**. Differentially expressed genes for relationship between PUFA and SFA. **Table S12**. Differentially expressed genes. **Table S13**. Differentially expressed genes for n6. **Table S14**. Differentially expressed genes for the ratio of ω6/ω3. (XLS 486 kb)

## Abbreviations

CLA: Conjugated linoleic acid
DEG: Differentially expressed genes
FA: Fatty acids
GO: Gene ontology
HIGH: The group with highest phenotype
LOW: The group with lowest phenotype
MUFA: Monounsaturated fatty acids
PUFA: Polyunsaturated fatty acids
SFA: Saturated fatty acids
ω3: Omega 3
ω6: Omega 6

## References

[1] Daley CA, Abbott A, Doyle PS, Nader GA, Larson S (2010). A review of fatty acid profiles and antioxidant content in grass-feed and grain-fed beef. Nutr J.

[2] Brugiapaglia A, Lussiana C, Destefanis G (2014). Fatty acid profile and cholesterol content of beef at retail of Piemontese, Limousin and Friesian breeds. Meat Sci.

[3] Wood JD, Enser M, Fisher AV, Nute GR, Sheard PR, Richardson RI, Hughes SI, Whittington FM (2008). Fat deposition, fatty acid composition and meat quality: a review. Meat Sci.

[4] French P, O' Riordan EG, Monahan FJ (2000). Meat quality of steers finished on autumn grass, grass silage or concentrate-based diets. Meat Sci.

[5] Cook ME, Whigham LD, Yang M (2001). CLA inhibits the induction of prostaglandin and leukotriene synthesis. A natural substitute for non-steroidal anti-inflammatory drugs. International Conference on CLA.

[6] Varela A, Oliete B, Moreno T, Portela C, Monserrrat L, Carballo JA, Sánchez L (2004). Effect of pasture finishing on the meat characteristics and intramuscular fatty acid profile of steers of the Rubia Gallega breed. Meat Sci.

[7] Stables MJ, Gilroy DW (2011). Old and new generation lipid mediators in acute inflammation and resolution. Prog Lipid Res.

[8] Lawrence GD (2013). Dietary fats and health: dietary recommendations in the context of scientific evidence. Adv Nutr.

[9] Huerta-Leidenz NO, Cross HR, Savell JW, Lunt DK, Baker JF, Pelton LS, Smith B (1993). Comparison of the fatty acid composition of subcutaneous adipose tissue from mature Brahman and Hereford cows. J Anim Sci.

[10] Huerta-Leidenz NO, Cross HR, Saveli JW, Lunt DK, Baker LS, Smith B (1996). Fatty acid composition of subcutaneous adipose tissue from male calves at different stages of growth. J Anim Sci.

[11] Perry D, Nicholls PJ, Thompson JM (1998). The effect of sire breed on the melting point and fatty acid composition of subcutaneous fat in steers. J Anim Sci.

[12] Menezes LFG, Restle J, Brondani IL, Kozloski GV, Deschamps F, Sachet RH (2009). Perfil de ácidos graxos na carne de novilhos Charolês e Nelore puros e de gerações avançadas do cruzamento rotativo, terminados em confinamento. Cienc Rural.

[13] Rossato LV, Bressan MC, Rodrigues EC, Gama LT, Bessa RJB, Alves SPA (2010). Parâmetros físico-químicos e perfil de ácidos graxos da carne de bovinos Angus e Nelore terminados em pastagem. Revista Brasileiro Zootecnia.

[14] Bressan MC, Rossato LV, Rodrigues EC, Alves SP, Bessa RJ, Ramos EM, Gama LT (2011). Genotype × environment interactions for fatty acid profiles in *Bos indicus* and *Bos taurus* finished on pasture or grain. J Anim Sci.

[15] Taniguchi M, Guan LL, Zhang B, Dodson MV, Okine E, Moore SS (2008). Gene expression patterns of bovine perimuscular preadipocytes during adipogenesis. Biochem Biophys Res Commun.

[16] Mannen H (2011). Identification and utilization of genes associated with beef qualities. Anim Sci J.

[17] Bauchart D (1993). Lipid absorption and transport in ruminants. J Dairy Sci.

[18] Chilliard Y (1993). Dietary fat and adipose tissue metabolism in ruminants, pigs, and rodents: a review. J Dairy Sci.

[19] Jenkins TC (1993). Lipid metabolism in the rumen, review. J Dairy Sci.

[20] Laliotis GP, Bizelis I, Rogdakis E (2010). Comparative approach of the de novo fatty acid synthesis (Lipogenesis) between ruminant and non ruminant mammalian species: from biochemical level to the main regulatory lipogenic genes. Curr Genomics.

[21] Ekine-Dzivenu C, Chen L, Vinsky M, Aldai N, Dugan MER, Mcallister TA, Wang Z, Okine E, Li C (2014). Estimates of genetic parameters for fatty acids in brisket adipose tissue of Canadian commercial crossbred beef steers. Meat Sci.

[22] Ramayo-Caldas Y, Mercadé A, Castelló A, Yang B, Rodríguez C, Alves E, Días I, Ibáñez-Escriche N, Noguera JL, Pérez-Enciso M, Fernández AL, Folch JM (2012). Genome-wide association study for intramuscular fatty acid composition in an Iberian x Landrace cross. J Anim Sci.

[23] Costa P, Lemos JP, Lopes PA, Alfaia CM, Costa AS, Bessa RJ, Prates JA (2012). Effect of low- and high-forage diets on meat quality and fatty acid composition of Alentejana and Barrosã beef breeds. Animal.

[24] Buchanan JW, Garmyn AJ, Hilton GG, Vanoverbeke DL, Beitz QDDC, Mateescu RG (2013). Comparison of gene expression and fatty acid profiles in concentrate and forage finished beef. J Anim Sci.

[25] Ferraz JBS, De Felício P (2010). Production systems - an example from Brazil. Meat Sci.

[26] Bligh EG, Dyer WJ (1959). A rapid method of total lipid extraction and purification. Can J Biochem Physiol.

[27] Folch J, Lees M, Sloane-Stanley GH (1957). A simple method for the isolation and purification of lipids from animal tissues. J Biol Chem.

[28] Kramer JKG, Fellner V, Dugan MER, Sauer FD, Mossoba MM, Yurawecz MP (1997). Evaluating acid and base catalysts in the methylation of milk and rumen and rumen fatty acids with special emphasis on conjugated dienes and total trans fatty acids. Lipids.

[29] Trapnell C, Roberts A, Goff L, Pertea G, Kim D, Kelley DR (2012). Differential gene and transcript expression analysis of RNA-seq experiments with TopHat and Cufflinks. Nat Protoc.

[30] Goff SA, Vaughn M, Mckay S, Lyons E (2011). The iPlant collaborative: cyberinfrastructure for plant biology. Front Plant Sci.

[31] Huang DW, Sherman BT, Lempicki RA (2009). Systematic and integrative analysis of large gene lists using DAVID Bioinformatics resources. Nat Protoc.

[32] Huang DW, Sherman BT, Lempicki RA (2009). Bioinformatics enrichment tools: paths toward the comprehensive functional analysis of large gene lists. Nucleic Acids Res.

[33] Hardie DG (2012). Organismal carbohydrate and lipid homeostasis. Cold Spring Harb Perspect Biol.

[34] Prado JM, Prado IN, Visentainer JV, et al. The effect of breed on the chemical composition and fatty acid profile of the *Longissimus dorsi* muscle of Brazilian beef cattle. J Anim Feed Sci. 2009;18:231–40.

[35] Cesar ASM, Regitano LCA, Mouão GB, Tullio RR, Lanna DPD (2014). Genome-wide association study for intramuscular fat deposition and composition in Nellore cattle. BMC Genet.

[36] Vernon RG, Flint DJ (1983). Proc Nutr Soc.

[37] Vernon RG, Flint DJ (1988). Proc Nutr Soc.

[38] Polizel Neto A, Branco RH, Bonilha SFM, Gomes HFB, Corvino TLS. Papel dos Ácidos Graxos Voláteis na Deposição de Tecido Adiposo Intramuscular – Revisão. 2008. http://www.infobibos.com/Artigos/2008_3/AcidosGraxos/index.htm. Accessed 17 Dec 2014.

[39] Ito RH, Prado IN, Rotta PP, Oliveira MG, Prado RM, Moletta JL (2012). Carcass characteristics, chemical composition and fatty acids profile of *longissimus* muscle of young bulls from four genetic groups finished in feedlot. Revista Brasileira de Zootecnia.

[40] Xie YR, Busboom JR, Gaskins CT, Johnson KA, Reeves JJ, Wright RW, Cronrath JD (1996). Effects of breed and sire on carcass characteristics and fatty acid profiles of crossbred Wagyu and Angus steers. Meat Sci.

[41] Warren HE, Scollan ND, Enser M, Hughes SI, Richardson RI, Wood JD (2008). Effects of breed and a concentrate or grass silage diet on beef quality in cattle of 3 ages. I: Animal performance, carcass quality and muscle fatty acid composition. Meat Sci.

[42] Jeong J, Kwon EG, Im SK, Seo KS, Baik M (2012). Expression of fat deposition and fat removal genes is associated with intramuscular fat content in *longissimus dorsi* muscle of Korean cattle steers. J Anim Sci.

[43] Wang H, Eckel RH (2009). Lipoprotein lipase: from gene to obesity. Am J Physiol Endocrinol Metab.

[44] Vernon RG, Finley E, Taylor E, Flint DJ (1985). Insulin binding and action of bovine adipocytes. Endocrinology.

[45] Miller JR, Thomsen PD, Dixon SC, Tucker EM, Konfortov BA, Harbitz I (1991). Synteny mapping of the bovine IGHG2, CRC and IGF-1 genes. Anim Genet.

[46] Rhoades RD, Sawyer JE, Chung KY, Schell ML, Lunt DK, Smith SB (2007). Effect of dietary energy source on in vitro substrate utilization and insulin sensitivity of muscle and adipose tissues of Angus and Wagyu steers. J Anim Sci.

[47] Rhoades RD, Sawyer JE, Ponce CH, Lunt DK, Smith SB (2009). Substrate utilization and dose response to insulin by subcutaneous adipose tissue of Angus steers fed corn- or hay-based diets. J Anim Sci.

[48] De Smet S, Raes K, Demeyer D (2004). Meat fatty acid composition as affected by fatness and genetic factors: a review. Animal Research.

[49] Muers M (2013). Sequencing for disease architecture. Nat Rev Genet.

[50] Chen ZJ, Zhao H, He L, Shi Y, Qin Y (2011). Genome-wide association study identifies susceptibility loci for polycystic ovary syndrome on chromosome. Nat Genet.

[51] Tizioto PC, Coutinho LL, Decker JE, Schnabel RD, Rosa KO (2015). Global liver gene expression differences in Nelore steers with divergent residual feed intake phenotypes. BMC Genomics.

[52] Lemos MVA, Chiaia HLJ, Berton MP, Feitosa FLB, Aboujaoude C (2016). Genome-wide association between single nucleotide polymorphisms with beef fatty acid profile in Nellore cattle using the single step procedure. BMC Genomics.

[53] Calder PC (2011). Fatty acids and inflammation: the cutting edge between food and pharma. Eur J Pharmacol.

[54] Cui HX, Liu RR, Zhao GP, Zheng MQ, Chen JL (2012). Identification of differentially expressed genes and pathways for intramuscular fat deposition in pectoralis major tissues of fast-and slow-growing chickens. BMC Genomics.

[55] Jiang S, Wei H, Song T, Yang Y, Peng J, Jiang S (2013). Transcriptome comparison between porcine subcutaneous and intramuscular stromal vascular cells during adipogenic differentiation. PLoS One.

[56] Cesar ASM, Regitano LC, Koltes JE, Fritz-Waters ER, Lanna DP, Gasparin G (2015). Putative regulatory factors associated with intramuscular fat content. PLoS One.

[57] Lee H-J, Mi J, Kim H, Kwark M, et al. Comparative transcriptome analysis of adipose tissues reveals that ECM-receptor interaction is involved in the depot-specific adipogenesis in cattle. PLoS One. 2013. doi:10.1371/journal.pone.0066267 DOI:10.1371%2Fjournal.pone.0066267#pmc_ext. 10.1371/journal.pone.0066267 PMC3689780 23805208

[58] Lee HJ, Park HS, Kim W, Yoon D, Seo S (2014). Comparison of metabolic network between muscle and intramuscular adipose tissues in Hanwoo beef cattle using a systems biology approach. Int J Genomics.

[59] Cánovas A, Varona L, Burgos C, Galve A, Carrodeguas JA, Ibáñez-Escriche N, Martín-Burriel I, López-Buesa P (2012). Early postmortem gene expression and its relationship to composition and quality traits in pig Longissimus dorsi muscle. J Anim Sci.

[60] Mehla K, Magotra A, Choudhary J, Singh AK (2014). Genome-wide analysis of the heat stress response in Zebu (Sahiwal) cattle. Gene.

[61] Berger J, Moller De (2002). The mechanisms of action of PPARs. Annu Rev Med.

[62] Hihi AK, Michalik L, Wahli W (2002). PPARs: transcriptional effectors of fatty acids and their derivatives. Cell Mol Life Sci.

[63] Abbott BD (2009). Review of the expression of peroxisome proliferator-activated receptors alpha (PPAR alpha), beta (PPAR beta), and gamma (PPAR gamma) in rodent and human development. Reprod Toxicol.

[64] Ehrenborg E, Krook A (2009). Regulation of skeletal muscle physiology and metabolism by peroxisome proliferator-activated receptor delta. Pharmacol Rev.

[65] Kersten S, Desvergne B, Wahli W (2000). Roles of PPARs in health and disease. Nature.

[66] Kersten S (2008). Peroxisome proliferator activated receptors and lipoprotein metabolism. PPAR Res.

[67] Doran AG, Berry DP, Creevey CJ (2014). Whole genome association study identifies regions of the bovine genome and biological pathways involved in carcass trait performance in Holstein-Friesian cattle. BMC Genomics.

[68] He K, Wang Q, Wang Z, Pan Y (2013). Association study between gene polymorphisms in PPAR signaling pathway and porcine meat quality traits. Mamm Genome.

[69] MAPA – Ministério da Agricultura, Pecuária e Abastecimento (2000). Instrução Normativa n^o^3, de 17 de Janeiro de 2000 http://www.agricultura.gov.br/arq_editor/file/Ministerio/concursos/em_andamento/instrucoes%20normativas/INT%20003%2017%2001%202000%20ABATE%20HUMANIT%25C1RIO%20ANIMAIS%20DE%20ACOUGUE.doc.

[70] Kyoto Encyclopedia of Genes and Genomes. 2015. http://david.abcc.ncifcrf.gov/kegg.jsp?path=bta00650$Butanoate%20metabolism&termId=470015001&source=kegg. Accessed 13 Apr 2015.

[71] Kyoto Encyclopedia of Genes and Genomes. 2015. http://david.abcc.ncifcrf.gov/kegg.jsp?path=bta00620$Pyruvate%20metabolism&termId=470014998&source=kegg. Accessed 13 Apr 2015.

[72] Kyoto Encyclopedia of Genes and Genomes. 2015 http://david.abcc.ncifcrf.gov/kegg.jsp?path=bta00561$Glycerolipid%20metabolism&termId=470014986&source=kegg. Accessed 13 Apr 2015.

[73] Kyoto Encyclopedia of Genes and Genomes. 2015. http://david.abcc.ncifcrf.gov/kegg.jsp?path=bta00072$Synthesis%20and%20degradation%20of%20ketone%20bodies&termId=470014947&source=kegg. Accessed 13 Apr 2015.

