# Supplementary material for: Gene expression profile of intramuscular muscle in Nellore cattle with extreme values of fatty acid
Source: BMC Genomics. 2016 Nov 25;17:972. doi: 10.1186/s12864-016-3232-y (PMC5123393; doi:10.1186/s12864-016-3232-y)
Supplement: Additional file 1: — Boxplot of the transcript abundance distribution for each beef fatty acid and gene expressed. (PDF 42 kb) [file 12864_2016_3232_MOESM1_ESM.pdf]

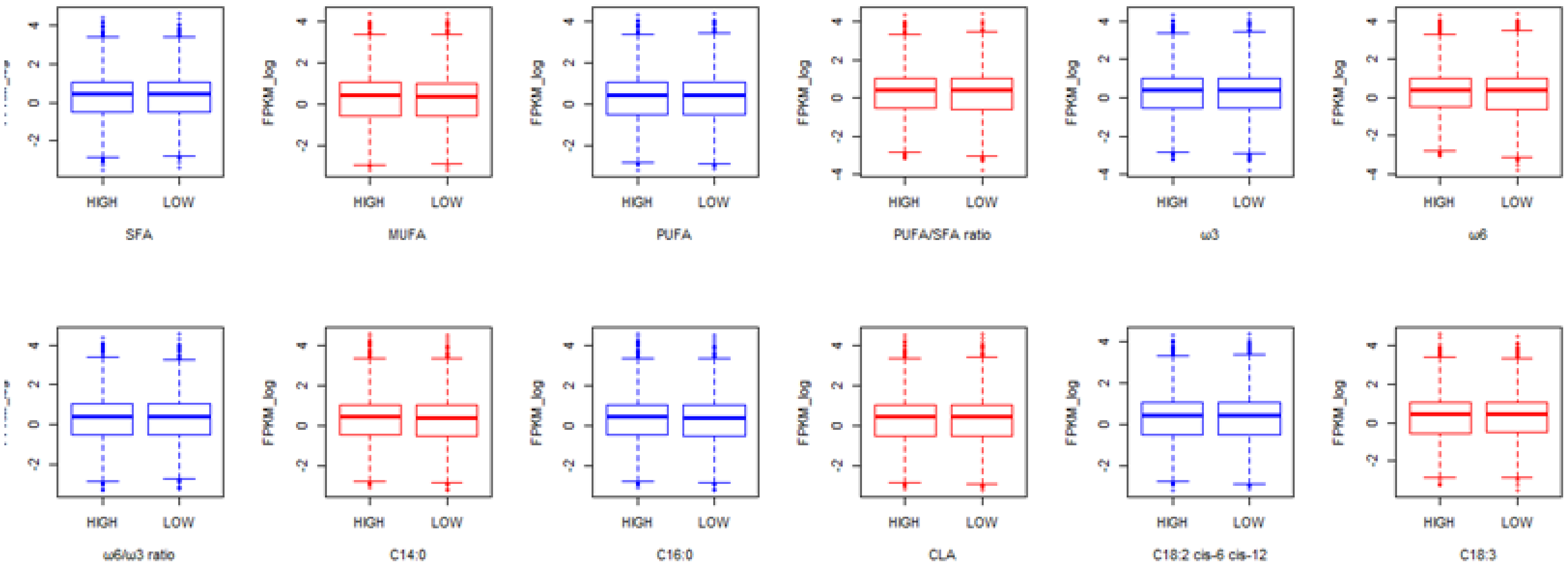

Additional File 1. Boxplot of the transcript abundance distribution for each beef fatty acid and gene expressed.
