# Supplementary material for: Gene expression profile of intramuscular muscle in Nellore cattle with extreme values of fatty acid
Source: BMC Genomics. 2016 Nov 25;17:972. doi: 10.1186/s12864-016-3232-y (PMC5123393; doi:10.1186/s12864-016-3232-y)
Supplement: Additional file 2: — Principal Component Analysis (PCA) of gene FPKM values between the high and low groups with fatty acids profile: (a) PCA for sum of SFA; (b) PCA for sum of MUFA; (c) PCA for sum of PUFA; (d) PCA for sum of PUFA/SFA ratio; (e) PCA for sum of n3;.(f) PCA for sum of n6; (g) PCA for n6/n3 ratio;(h) PCA for myristic acid (C14:0); (i) PCA for palmitic acid (C16:0); (j) PCA for stearic acid (C18:0);(k) PCA for oleic acid (C18:1 cis-9); (l) PCA for conjugated linoleic acid (C18:2 cis-9 trans-11); (m) PCA for linoleic acid (C18:2 cis6 cis12 n6); (n) PCA for α-linolenic (C18:3 n3). (PDF 459 kb) [file 12864_2016_3232_MOESM2_ESM.pdf]

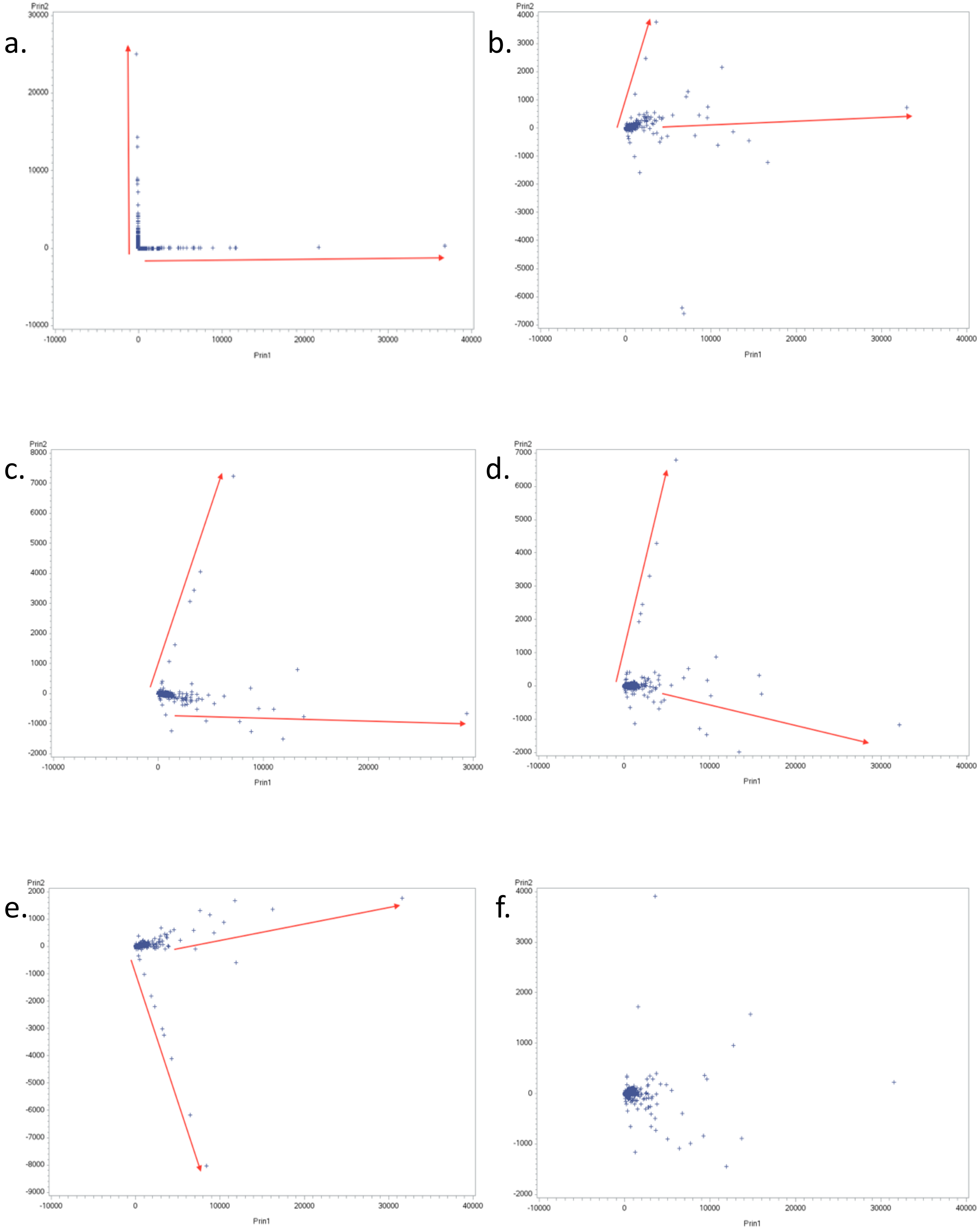
a.
Prin2
30000
20000
10000
0
-10000
-10000
0
10000
20000
30000
40000
Prin1
b.
Prin2
4000
3000
2000
1000
0
-1000
-2000
-3000
-4000
-5000
-6000
-7000
-10000
0
10000
20000
30000
40000
Prin1
c.
Prin2
8000
7000
6000
5000
4000
3000
2000
1000
0
-1000
-2000
-10000
0
10000
20000
30000
Prin1
d.
Prin2
7000
6000
5000
4000
3000
2000
1000
0
-1000
-2000
-10000
0
10000
20000
30000
40000
Prin1
e.
Prin2
2000
1000
0
-1000
-2000
-3000
-4000
-5000
-6000
-7000
-8000
-9000
-10000
0
10000
20000
30000
40000
Prin1
f.
Prin2
4000
3000
2000
1000
0
-1000
-2000
-10000
0
10000
20000
30000
40000
Prin1

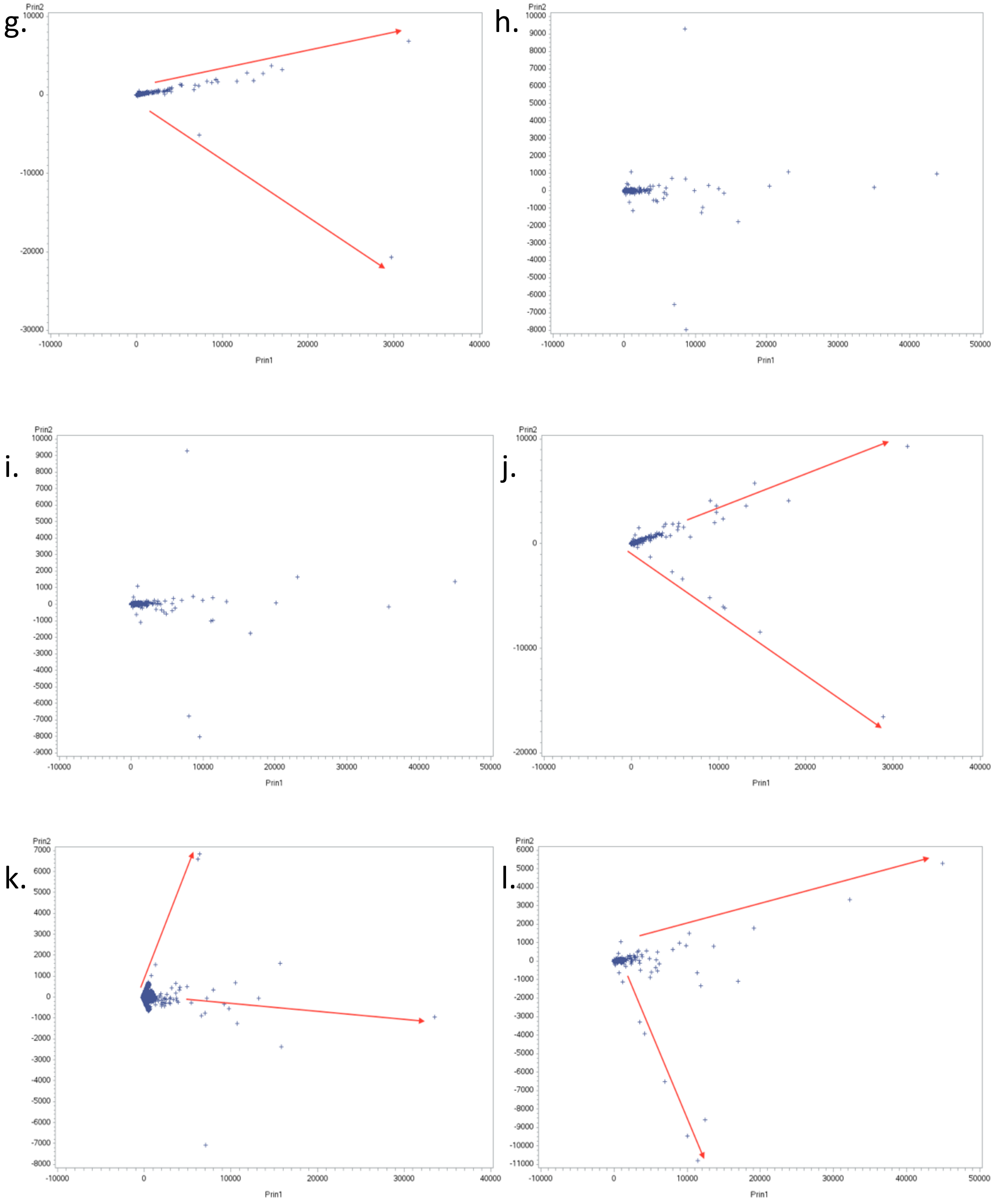
g.
h.
i.
j.
k.
l.
Prin2
Prin1

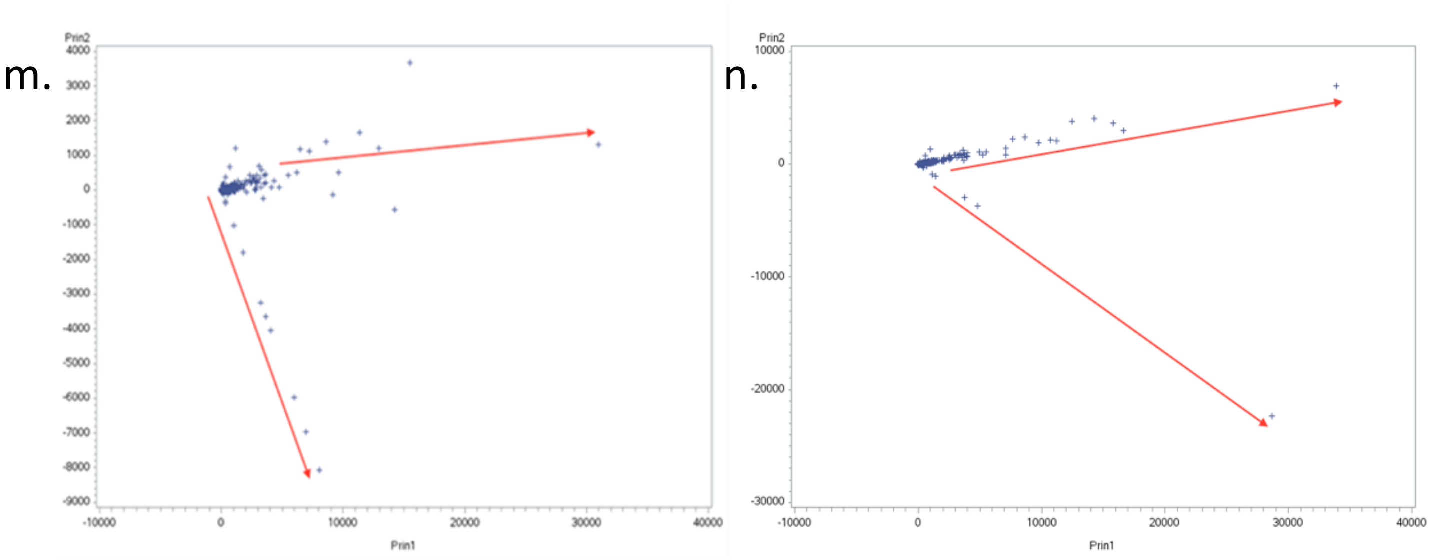

Additional File 2. Principal Component Analysis (PCA) of gene FPKM values between the high and low groups with fatty acids profile: (a) PCA for sum of SFA; (b) PCA for sum of MUFA; (c) PCA for sum of PUFA; (d) PCA for sum of PUFA/SFA ratio; (e) PCA for sum of n3;.(f) PCA for sum of n6; (g) PCA for n6/n3 ratio;(h) PCA for myristic acid (C14:0); (i) PCA for palmitic acid (C16:0); (j) PCA for stearic acid (C18:0);(k) PCA for oleic acid (C18:1 cis-9); (l) PCA for conjugated linoleic acid (C18:2 cis-9 trans-11); (m) PCA for linoleic acid (C18:2 cis6 cis12 n6); (n) PCA for α-linolenic (C18:3 n3).
